# Dysregulated Ca^2+^-Permeable AMPA Receptor Signaling in Neural Progenitors Modeling Fragile X Syndrome

**DOI:** 10.3389/fnsyn.2019.00002

**Published:** 2019-02-08

**Authors:** Claudia Danesi, Kari Keinänen, Maija L. Castrén

**Affiliations:** ^1^Department of Physiology, Faculty of Medicine, University of Helsinki, Helsinki, Finland; ^2^Research Program in Molecular and Integrative Biosciences, Faculty of Biological and Environmental Sciences, University of Helsinki, Helsinki, Finland

**Keywords:** autism, AMPA, GluA2, fragile X syndrome, miRNA, miR-181, neural progenitor, plasticity

## Abstract

Fragile X syndrome (FXS) is a neurodevelopmental disorder that represents a common cause of intellectual disability and is a variant of autism spectrum disorder (ASD). Studies that have searched for similarities in syndromic and non-syndromic forms of ASD have paid special attention to alterations of maturation and function of glutamatergic synapses. Copy number variations (CNVs) in the loci containing genes encoding alpha-amino-3-hydroxy-5-methylisoxazole-4-propionic acid receptors (AMPARs) subunits are associated with ASD in genetic studies. In FXS, dysregulated AMPAR subunit expression and trafficking affect neural progenitor differentiation and synapse formation and neuronal plasticity in the mature brain. Decreased expression of GluA2, the AMPAR subunit that critically controls Ca^2+^-permeability, and a concomitant increase in Ca^2+^-permeable AMPARs (CP-AMPARs) in human and mouse FXS neural progenitors parallels changes in expression of GluA2-targeting microRNAs (miRNAs). Thus, posttranscriptional regulation of GluA2 by miRNAs and subsequent alterations in calcium signaling may contribute to abnormal synaptic function in FXS and, by implication, in some forms of ASD.

## Introduction

Autism spectrum disorder (ASD) is a heterogeneous group of neurodevelopmental disorders that are characterized by defective social interaction, impairment in verbal and nonverbal communication, repetitive and restricted behavior, and sensory abnormalities (American Psychiatric Association, 2013). Many autistic individuals display a variety of additional neuropsychiatric features (Simonoff et al., 2008), an abnormal intellectual profile (Fombonne, 2003; Rydzewska et al., 2018), and epilepsy (Besag, 2017), indicating a high rate of co-morbidity among the neurodevelopmental defects (DiCicco-Bloom et al., 2006). ASD has a strong genetic component and genetic studies have implicated hundreds of genes associated with increased risk of ASD (Persico and Napolioni, 2013). Extreme locus heterogeneity in ASD suggests that an interplay of common and rare genetic variations contribute to the ASD phenotype (O’Roak et al., 2012). These findings have led to the identification of candidate pathways and functional changes involved in the pathophysiology of ASD (Pinto et al., 2010). Many of the ASD risk genes are particularly important during brain development (Pardo and Eberhart, 2007). Features of autism associate with distinct rare monogenic neurodevelopmental syndromes, including fragile X syndrome (FXS), tuberosis sclerosis, and Rett syndrome (Lintas et al., 2012). Syndromic single-gene disorders provide an excellent possibility to investigate the molecular and cellular mechanisms that increase the risk of autism. In this context, maturation and function of glutamatergic synapses have received attention (Uzunova et al., 2014; Fung and Hardan, 2015).

Alpha-amino-3-hydroxy-5-methylisoxazole-4-propionic acid receptors (AMPARs) are the main mediators of excitatory transmission and synaptic strength in neuronal plasticity. A decreased density of AMPARs has been found in the post-mortem cerebellum of individuals with autism (Purcell et al., 2001). Furthermore, AMPAR modulation can normalize abnormal excitatory transmission and social impairments in animal models of ASD (Kim et al., 2019). AMPARs are formed by four subunits, GluA1–4. The predominantly expressed edited GluA2 subunit makes the receptor impermeable to divalent cations, whereas the receptors lacking GluA2 are permeable to Ca^2+^ and show strong inward rectification that is caused by intracellular polyamine block (Donevan and Rogawski, 1995; Bowie et al., 1998; Kumar et al., 2002). A genetic deletion that includes the *GRIA2* gene encoding GluA2 is associated with autism (Ramanathan et al., 2004) and mutations of RAB39B that cause intellectual disability comorbid with autism lead to impaired transport of GluA2 to synapses and subsequent shift of AMPAR to higher calcium permeability (Mignogna et al., 2015).

FXS is the most common inherited intellectual disability syndrome (Santoro et al., 2012) and is considered as the most common single-gene condition associated with autism (Hernandez et al., 2009). The FXS phenotype includes hyperactivity, defects in sensory integration, communication difficulties, poor motor coordination, social anxiety, and restricted repetitive and stereotyped patterns of behavior (Hagerman et al., 2010). Epilepsy in 13%–44% of FXS cases shows an age-related appearance (Kluger et al., 1996; Berry-Kravis, 2002; Louhivuori et al., 2009). FXS accounts for 5%–7% of all ASD cases (Hagerman et al., 2010). Depending on the diagnostic criteria used, 30%–54% of males and 16%–20% of females with FXS fulfil the standardized criteria of autism (Brown et al., 1986; Hernandez et al., 2009; Kaufmann et al., 2017). Impaired glutamate receptor-mediated plasticity is implicated in FXS and an imbalance of excitation and inhibition at the neuronal circuit level has been found in the mouse model of FXS [*Fmr1* knockout (KO) mice; Bear et al., 2004; Bassel and Warren, 2008; Gibson et al., 2008; Harlow et al., 2010; Gonçalves et al., 2013; Zhang et al., 2014]. This review summarizes AMPAR alterations observed in FXS used as a model for studies of autism symptomatology.

## Altered Synapse Function in FXS

FXS is caused by the absence of the FMR1 protein (FMRP), which results from promoter methylation and transcriptional silencing of the *FMR1* gene with CGG triplet repeat expansion (>200 repeats) in the 5′ untranslated part of the gene (Verkerk et al., 1991). FMRP is an RNA-binding protein that is involved in the regulation of transport and translation of specific mRNAs. It is estimated that roughly 4% of brain mRNAs interacts with FMRP (Brown et al., 2001) and many of the FMRP target mRNAs are associated with ASD (Darnell et al., 2011). In addition to mRNA interactions, FMRP associates with microRNAs (miRNAs) and components of the miRNA pathway, including Dicer and Argonaute proteins (Siew et al., 2013). In addition, direct protein-protein interactions between FMRP and ion channels have been found (Brown et al., 2010; Ferron, 2016). Several presynaptic and postsynaptic proteins, including proteins involved in the regulation of membrane excitability, ionic homeostasis, and neurotransmitter release, are abnormally regulated in the brain of *Fmr1* KO mice, which recapitulate the main human FXS phenotype (Jin and Warren, 2000).

Exaggerated type I metabotropic glutamate receptor (mGluR)-mediated synaptic translation enhances long-term synaptic depression (LTD) in the absence of FMRP (Huber et al., 2002; Bear et al., 2004). Long-term potentiation (LTP) is also affected in *Fmr1* KO mice (Li et al., 2002; Zhao et al., 2005; Desai et al., 2006; Lauterborn et al., 2007; Wang et al., 2010; Xu et al., 2012). Differences in the threshold for the induction of LTP in the absence of FMRP implicate altered neuronal and particularly dendritic excitability to neuronal plasticity changes (Meredith et al., 2007; Meredith and Mansvelder, 2010). Compromised spike-timing-dependent LTP in the prefrontal cortex of *Fmr1* KO mice can be restored by increasing neuronal activity and rearing these mice in an enriched environment restores synaptic plasticity (Meredith et al., 2007). There is evidence that the absence of FMRP leads to dysregulation of several ion channels, including L-type voltage-gated calcium channels (Meredith et al., 2007; Castagnola et al., 2018; Danesi et al., 2018) and potassium (K+) channels (Brown et al., 2010; Deng et al., 2013), which may contribute to defects in cellular excitability and neuronal plasticity in FXS.

## Dysregulated Localization of AMPAR in FXS

Ionotropic glutamate receptors are expressed already before synaptogenesis and have roles in neuronal development in addition to their function as mediators of synaptic transmission and plasticity in mature neurons (Schlett, 2006). Alterations of AMPARs are consistent with defective functional maturation and neuronal plasticity in the FXS mouse brain. In the neonatal brain, a large fraction of synapses are functionally silent due to the absence of AMPARs and tonic Mg^2+^ block of N-Methyl-D-aspartate receptors (NMDARs; Malenka and Nicoll, 1997; Hanse et al., 2013). Upon later development, insertion of AMPARs to synaptic membranes unsilences most of the silent synapses (Wu et al., 1996). Silent synapses are increased in the *Fmr1* KO mouse brain during the first postnatal weeks (Harlow et al., 2010). The AMPA/NMDA amplitude ratio of evoked synaptic responses appears to be the lowest before closure of the critical period, reflecting delayed synapse stabilization which involves delayed refinement of cortical excitatory circuits in the FXS mouse brain (Harlow et al., 2010).

FMRP promotes neuronal maturation by membrane delivery of GluA1 without affecting total GluA1 protein expression (Darnell et al., 2011; Guo et al., 2015). In contrast, the autosomal paralog of FMRP, FXR2P, increases GluA1 expression *via* direct interaction with *GluA1* mRNA and affects mRNA stability (Guo et al., 2015). In the *Fmr1* KO mouse brain, internalization of surface GluA1 is abnormal in the prefrontal cortex and amygdala, which represent brain regions implicated in the neuropathology of autism (Suvrathan et al., 2010; Wang et al., 2010). Augmented LTD leads to increased AMPAR internalization in the hippocampus and cerebellum of the *Fmr1* KO mouse (Huber et al., 2002; Bear et al., 2004; Nakamoto et al., 2007). Retinoic acid-dependent synaptic scaling *via* dendritic translation of GluA1 subunit of AMPARs is also disturbed (Soden and Chen, 2010). Expression of GluA1 and GluA2 mRNAs is reduced in the prefrontal cortex of the *Fmr1* KO mouse when compared to wild-type controls (Achuta et al., 2018), but it is not known whether the changes of AMPAR expression observed in the FXS mouse brain correlate with changes in human brain.

## Ion Channel Function of AMPAR in FXS

Independent of their role in synaptic transmission, Ca^2+^-permeable AMPARs (CP-AMPARs) have developmental functions that emerged already early in evolution (Hirai et al., 2017). Expression of GluA2 is low in the early developmental stages, suggesting that Ca^2+^ influx through CP-AMPARs contributes to the regulation of neuronal and glial development (Kumar et al., 2002; Zonouzi et al., 2011; Lalanne et al., 2016; Szczurowska et al., 2016). In normal neuronal development, the CP-AMPARs expressed during early development are subsequently replaced by GluA2-containing, calcium-impermeable receptors, which dominate in the mature nervous system (Kumar et al., 2002). Interestingly, in some disorders of developmental origin, this subunit switch is delayed leading to an increased GluA1/GluA2 ratio or calcium permeability (Talos et al., 2006; Ruffolo et al., 2016), consistent with recent findings in studies performed on human FXS neural progenitors (Achuta et al., 2018). AMPARs are expressed in the embryonic proliferative ventricular and subventricular zones, where the neural progenitors reside (Lidow and Rakic, 1995; LoTurco et al., 1995). Neural progenitors express both AMPA and NMDA receptors in a progenitor type- and developmental stage-dependent manner, and these receptors mediate important regulatory signals during progenitor differentiation (LoTurco et al., 1995; Brazel et al., 2005; Jansson et al., 2011, 2013; Achuta et al., 2017). In the rat brain, GluA2-lacking AMPARs are expressed during the first postnatal week initially in radial glia (López et al., 1994) followed by an “inside-out” gradient in pre-oligodendrocytes and subplate neurons and later in cortical neurons (Talos et al., 2006). Oligodendrocyte progenitor cells (OPCs) express AMPA/kainate (KA) receptors and specific agonists of these receptors cause reversible G1 arrest of OPC cell cycle *via* Ca^2+^-independent means (Gallo et al., 1996). Ca^2+^ influx-mediated excitotoxic cell death is promoted by activation of AMPA/KA receptors in postmitotic pre-oligodendrocytes, whereas a similar sensitivity to excitotoxic insults is not observed in proliferating OPCs or mature myelinating oligodendrocytes.

When compared with normal controls at the early stage of progenitor differentiation, functional analysis has revealed enhanced intracellular Ca^2+^ responses to AMPAR activation in neural progenitors differentiated from human induced pluripotent stem (iPS) cells generated from somatic cells of FXS males (Achuta et al., 2018). Augmented CP-AMPAR responses associate with increased Ca^2+^ influx *via* L-type voltage-gated calcium channels and hyperresponsiveness to membrane depolarization and to NMDA and mGluR type I receptor activation (Danesi et al., 2018). Whitney et al. have shown that activation of CP-AMPARs induces neuronal differentiation in human neural progenitors. Consistent with these findings, differentiation of glutamate-responsive progenitors is enhanced from FXS progenitors (Whitney et al., 2008). Blocking CP-AMPARs also affects neurite outgrowth in both FXS and wild-type neural progenitors (Whitney et al., 2008; Achuta et al., 2018). The increase in CP-AMPARs correlates with an increased inward rectification and a reduced number of GluA2 subunit-expressing cells in mouse FXS progenitors. CP-AMPARs provide an important route for Ca^2+^ entry into progenitors, and changes of Ca^2+^ signaling can contribute to altered fate determination, differentiation, and migration of FXS neural progenitors (Castrén et al., 2005; Tervonen et al., 2009; Saffary and Xie, 2011; Sheridan et al., 2011; Liu et al., 2012; Doers et al., 2014; Li and Zhao, 2014; Telias et al., 2015). Increased Ca^2+^ influx through CP-AMPARs renders FXS neural progenitors more susceptible to excitotoxicity. Increased vulnerability may act as a selective factor during cell differentiation and may interfere with establishment of neocortical circuits leading to network hyperexcitability in the *Fmr1* KO mouse brain (Meredith et al., 2007, 2011). Since CP-AMPARs also influence morphological plasticity and migration of neurons, increased CP-AMPAR signaling may be involved in the delayed positioning of glutamatergic neurons to the cortical plate and abnormal morphological transformation of migrating cells in the developing cortex of the FXS mouse (La Fata et al., 2014).

The GluA2 subunit imparts Ca^2+^ -permeability to AMPARs only when it contains an arginine residue in a critical position (“Q/R site”) of the ion channel, which is introduced by RNA editing of the GluA2 primary transcript by adenosine deaminase ADAR2 (Sommer et al., 1991; Wong et al., 2001; Wright and Vissel, 2012). The GluA2 transcripts are nearly entirely edited in the adult brain (Wright and Vissel, 2012). During development, including in neural progenitors, editing of GluA2 transcripts is also very efficient, although ADAR2 expression levels are low (Pachernegg et al., 2015). Unedited Ca^2+^-permeable AMPARs are found under pathological conditions (Kwak and Kawahara, 2005; Peng et al., 2006). The *Drosophila* fragile X homolog (dFMR1) modulates activity of the RNA editing enzyme dADAR (Bhogal et al., 2011) and it was recently shown that FMRP promotes RNA editing in the human brain (Tran et al., 2019). FMRP regulation of RNA editing was identified as a common mechanism causing hypoediting of GRIA2 and GRIA4 in the human ASD and FXS brain (Tran et al., 2019).

## miRNA-Dependent Regulation of AMPAR

Numerous mammalian genes are targets of miRNAs (Lewis et al., 2005). These small (19–24 nucleotides in length) noncoding RNAs act as post-transcriptional regulators of mRNA translation and stability (Lee et al., 1993; Bartel, 2004). In the nervous system, miRNAs play important regulatory roles during synapse formation and in synaptic plasticity and memory formation (Fregeac et al., 2016). Several miRNAs are implicated in the etiology and pathogenesis of neurodegenerative, neurological, and neuropsychiatric disorders, including autism (Beveridge et al., 2010; Ghahramani Seno et al., 2011). A number of miRNAs, including miR-124 (Ho et al., 2014), miR-181 (Beveridge et al., 2010), miR-223 (Harraza et al., 2012), and miR-409 and miR-495 (Capauto et al., 2018) have been found to target GluA2 AMPAR subunit mRNA and therefore have the potential to regulate subunit composition and calcium-permeability of AMPAR.

**Figure 1:**
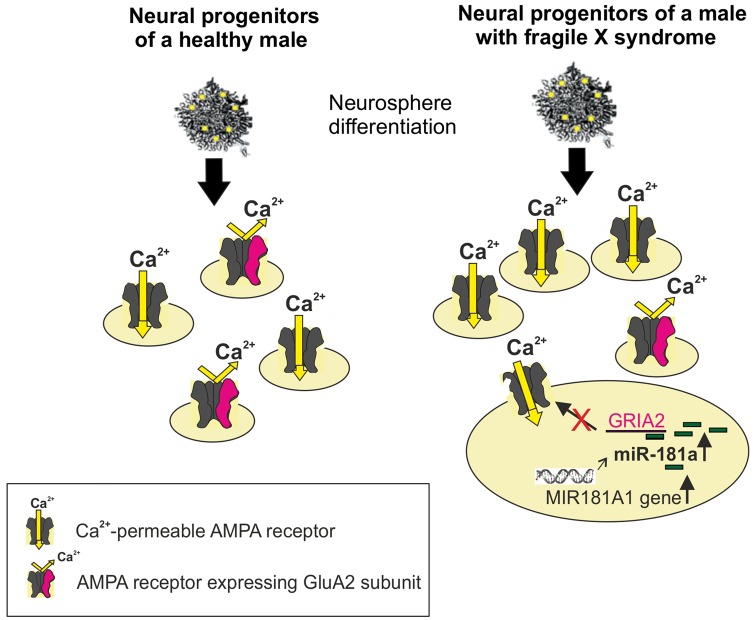
Schematic presentation of increased differentiation of neural progenitors expressing Ca^2+^-permeable (CP; alpha-amino-3-hydroxy-5-methylisoxazole-4-propionic acid receptor, AMPAR) from human Fragile X syndrome (FXS) induced pluripotent stem (iPS)-derived neurospheres compared with normal healthy controls. The miR-181a-mediated regulation of Ca^2+^-permeability of the AMPAR is visualized in a FXS progenitor by showing the increased expression of the *MIR181A1* gene. This leads to an increase in the expression of mature miR-181a, which by interacting with the *GRIA2* mRNA, can post-trascriptionally reduce the translation of the GluA2 subunit. The edited GluA2 subunit is required for Ca^2+^-impermeability of the AMPAR.

Reduced GluA2 protein expression correlates with increased expression of the noncoding* MIR-181A1* gene (the host gene of mature miRNAs) and mature *miR-181a* in FXS neural progenitors (Achuta et al., 2018; Figure 1). Increased expression of *miR-181a-5p* and *miR-181a-3p* was found in both human and mouse neural progenitors at days 1 and 7 of neurosphere differentiation. Expression of *miR-181b* was not detectable at these early differentiation timepoints, which is consistent with the low expression of *miR-181b* in the embryonic brain compared to that in the adult cortex (Hutchison et al., 2013). Neurospheres consist of mixed populations of neural progenitors and these studies did not elucidate *miR-181a* expression and its functional consequences in a cell type-specific manner.

The association of miR-181 species to the FXS gene family with neuropsychiatric phenotype/ASD (Stepniak et al., 2015) and a microduplication of chromosome 1q32.1 in the region comprising the *MIR-181A1* gene in a patient case with developmental delay and autistic features (Olson et al., 2012) support a role for miR-181 in FXS/ASD. Furthermore, expression of miR-181 is increased in lymphoblastoid cell lines of individuals with ASD (Ghahramani Seno et al., 2011) and in the superior temporal gyrus and the dorsolateral prefrontal cortex of individuals with schizophrenia (Beveridge et al., 2010). There is evidence that miR-181a is developmentally regulated and involved in cell-fate determination in the central nervous system (Hutchison et al., 2013). The target genes of the miR-181 family are implicated in regulation of developmental mechanisms (Saba et al., 2012), neurotrophin signaling, and axon guidance (Yang et al., 2014), consistent with a role of miR-181 in the neuropathology of neurodevelopmental disorders. Since expression of miRNAs is spatiotemporally regulated, the expression of the miR-181 family and other miRNAs that regulate GluA2 remain to be studied in detail in the mouse and human developing and mature FXS/ASD brain.

## Conclusions/Summary

Functional changes of AMPARs and increased Ca^2+^ influx through AMPARs play an important role in the neurobiology of FXS. AMPAR-mediated alterations may serve as common pathological mechanisms in FXS and ASD. This is supported by studies showing RNA hypoediting of AMPAR subunits in the human FXS and ASD brain (Tran et al., 2019) and alterations of AMPAR subunit expression and trafficking by genetic mutations that cause ASD (Mignogna et al., 2015). The contribution of miRNA-mediated regulation of CP-AMPAR signaling remains to be further explored.

CP-AMPARs are implicated as critical mediators of neuronal death in epilepsy, ischemia, traumatic brain injury, and neurodegenerative disorders (Pellegrini-Giampietro et al., 1992; Spaethling et al., 2008; Szczurowska et al., 2016; Whitehead et al., 2017). This emphasizes the importance of CP-AMPAR-dependent mechanisms in pathological processes in the brain and as a potential target for therapeutic intervention. Human iPS cell-derived neuronal cells offer possibilities to perform further studies in a cell type-dependent manner in the actual disease model. Many rescue strategies that target excessive protein synthesis show beneficial effects on synaptic function and behavioral phenotype in the FXS mouse model (Richter et al., 2015). However, appropriate pharmaceutical compounds have not shown sufficient efficacy in clinical trials, thus indicating need for new treatment strategies (Berry-Kravis et al., 2011; Erickson et al., 2017). Improved understanding of alterations of CP-AMPAR signaling and their relationship to the direct pathophysiological and manifold compensatory changes in FXS may provide new avenues for treatment and biomarker discovery in FXS.

## Author Contributions

MC wrote the first draft of the manuscript. All authors contributed to and have approved the final manuscript.

## Conflict of Interest Statement

The authors declare that the research was conducted in the absence of any commercial or financial relationships that could be construed as a potential conflict of interest.

## References

[B1] Achuta V. Grym H. Putkonen N. Louhivuori V. Kärkkäinen V. Koistinaho J.. (2017). Metabotropic glutamate receptor 5 responses dictate differentiation of neural progenitors to NMDA-responsive cells in fragile X syndrome. Dev. Neurobiol. 77, 438–453. 10.1002/dneu.22419 27411166

[B2] Achuta V. Möykkynen T. Peteri U.-K. Turconi G. Rivera C. Keinänen K.. (2018). Functional changes of AMPA responses in human induced pluripotent stem cell-derived neural progenitors in autism spectrum disorder. Sci. Signal. 11:eaan8784. 10.1126/scisignal.aan8784 29339535

[B3] American Psychiatric Association (2013). Diagnostic and Statistical Manual of Mental Disorders, 5th Edn. Text Rev., DSM-5. Washington, DC: American Psychiatric Association (APA).

[B4] Bartel D. (2004). MicroRNAs: genomics, biogenesis, mechanism, and function. Cell 116, 281–297. 10.1016/S0092-8674(04)00045-5 14744438

[B5] Bassel G. Warren S. (2008). Fragile X syndrome: loss of local mRNA regulation alters synaptic development and function. Neuron 60, 201–214. 10.1016/j.neuron.2008.10.004 18957214 PMC3691995

[B6] Bear M. Huber K. Warren S. (2004). The mGluR theory of fragile X mental retardation. Trends Neurosci. 27, 370–377. 10.1016/j.tins.2004.04.009 15219735

[B7] Berry-Kravis E. (2002). Epilepsy in fragile X syndrome. Dev. Med. Child Neurol. 44, 724–728. 10.1017/s0012162201002833 12418611

[B8] Berry-Kravis E. Knox A. Hervey C. (2011). Targeted treatments for fragile X syndrome. J. Neurodev. Disord. 3, 193–210. 10.1007/s11689-011-9074-7 21484200 PMC3261278

[B9] Besag F. (2017). Epilepsy in patients with autism: links, risks and treatment challenges. Neuropsychiatr. Dis. Treat. 14, 1–10. 10.2147/ndt.s120509 29296085 PMC5739118

[B10] Beveridge N. J. Gardiner E. Carrol A. P. Tooney P. A. Cairns M. J. (2010). Schizophrenia is associated with an increase in cortical microRNA biogenesis. Mol. Psychiatry 15, 1176–1189. 10.1038/mp.2009.84 19721432 PMC2990188

[B11] Bhogal B. Jepson J. Savva Y. Pepper A. Reenan R. Jongens T. (2011). Modulation of dADAR-dependent RNA editing by the *Drosophila* fragile X mental retardation protein. Nat. Neurosci. 14, 1517–1524. 10.1038/nn.2950 22037499 PMC3225737

[B12] Bowie D. Lange G. Mayer M. (1998). Activity-dependent modulation of glutamate receptors by polyamines. J. Neurosci. 18, 8175–8185. 10.1523/JNEUROSCI.18-20-08175.1998 9763464 PMC6792845

[B13] Brazel C. Y. Nuñez J. L. Yang Z. Levison S. W. (2005). Glutamate enhances survival and proliferation of neural progenitors derived from the subventricular zone. Neuroscience 131, 55–65. 10.1016/j.neuroscience.2004.10.038 15680691

[B15] Brown W. Jenkins E. Cohen I. L. Fisch G. Wolf-Schein E. Gross A.. (1986). Fragile X and autism: a multicenter survey. Am. J. Med. Genet. 23, 341–352. 10.1002/ajmg.1320230126 3513570

[B16] Brown V. Jin P. Ceman S. Darnell J. O’Donnell W. Tenenbaum S.. (2001). Microarray identification of FMRP-associated brain mRNAs and altered mRNA translational profiles in fragile X syndrome. Cell 107, 477–487. 10.1016/s0092-8674(01)00568-2 11719188

[B14] Brown M. Kronengold J. Gazula V. Chen Y. Strumbos J. Sigworth F.. (2010). Fragile X mental retardation protein controls gating of the sodium-activated potassium channel Slack. Nat. Neurosci. 13, 819–821. 10.1038/nn.2563 20512134 PMC2893252

[B17] Capauto D. Colantoni A. Lu L. Santini T. Peruzzi G. Biscarini S.. (2018). A regulatory circuitry between Gria2, miR-409, and miR-495 is affected by ALS FUS mutation in ESC-derived motor neurons. Mol. Neurobiol. 55, 7635–7651. 10.1007/s12035-018-0884-4 29430619 PMC6132778

[B18] Castagnola S. Delhaye S. Folci A. Paquet A. Brau F. Duprat F.. (2018). New insights into the role of Ca_v_2 protein family in calcium flux Deregulation in *Fmr1*-KO neurons. Front. Mol. Neurosci. 11:342. 10.3389/fnmol.2018.00342 30319351 PMC6170614

[B19] Castrén M. Tervonen T. Karkkainen V. Heinonen S. Castrén E. Larsson K.. (2005). Altered differentiation of neural stem cells in fragile X syndrome. Proc. Natl. Acad. Sci. U S A 102, 17834–17839. 10.1073/pnas.0508995102 16314562 PMC1308923

[B20] Danesi D. Achuta V. Corcoran P. Peteri U.-K. Turconi G. Matsui N.. (2018). Increased calcium influx through L-type calcium channels in human and mouse neural progenitors lacking fragile X mental retardation protein. Stem Cell Reports 11, 1449–1461. 10.1016/j.stemcr.2018.11.003 30503263 PMC6294261

[B21] Darnell J. Van Driesche S. Zhang C. Hung K. Y. Mele A. Fraser C. E.. (2011). FMRP stalls ribosomal translocation on mRNAs linked to synaptic function and autism. Cell 146, 247–261. 10.1016/j.cell.2011.06.013 21784246 PMC3232425

[B22] Deng P. Rotman Z. Blundon J. Cho Y. Cui J. Cavalli V.. (2013). FMRP regulates neurotransmitter release and synaptic information transmission by modulating action potential duration via BK channels. Neuron 77, 696–711. 10.1016/j.neuron.2012.12.018 23439122 PMC3584349

[B23] Desai N. Casimiro T. Gruber S. Vanderklish P. (2006). Early postnatal plasticity in neocortex of *Fmr1* knockout mice. J. Neurophysiol. 96, 1734–1745. 10.1152/jn.00221.2006 16823030

[B24] DiCicco-Bloom E. Lord C. Zwaigenbaum L. Courchesne E. Dager S. Schmitz C.. (2006). The developmental neurobiology of autism spectrum disorder. J. Neurosci. 26, 6897–6906. 10.1523/JNEUROSCI.1712-06.2006 16807320 PMC6673916

[B25] Doers M. E. Musser M. T. Nichol R. Berndt E. R. Baker M. Gomez T. M.. (2014). iPSC-derived forebrain neurons from FXS individuals show defects in initial neurite outgrowth. Stem Cells Dev. 23, 1777–1787. 10.1089/scd.2014.0030 24654675 PMC4103262

[B26] Donevan S. Rogawski M. (1995). Intracellular polyamines mediate inward rectification of Ca^2+^-permeable α-amino-3-hydroxy-5-methyl-4-isoxazolepropionic acid receptors. Proc. Natl. Acad. Sci. U S A 92, 9298–9302. 10.1073/pnas.92.20.9298 7568121 PMC40972

[B27] Erickson C. Davenport M. Schaefer T. Wink L. Pedapati E. Sweeney J.. (2017). Fragile X targeted pharmacotherapy: lessons learned and future directions. J. Neurodev. Disord. 9:7. 10.1186/s11689-017-9186-9 28616096 PMC5467059

[B28] Ferron L. (2016). Fragile X mental retardation protein controls ion channel expression and activity. J. Physiol. 594, 5861–5867. 10.1113/jp270675 26864773 PMC5063927

[B29] Fombonne E. (2003). Epidemiological surveys of autism and other pervasive developmental disorders: an update. J. Autism Dev. Disord. 33, 365–382. 10.1023/A:1025054610557 12959416

[B30] Fregeac J. Colleaux L. Nguyen L. S. (2016). The emerging roles of MicroRNAs in autism spectrum disorders. Neurosci. Biobehav. Rev. 71, 729–738. 10.1016/j.neubiorev.2016.10.018 27793596

[B31] Fung L. Hardan A. (2015). Developing medications targeting glutamatergic dysfunction in autism: progress to date. CNS Drugs 29, 453–463. 10.1007/s40263-015-0252-0 26104862 PMC4515163

[B32] Gallo V. Zhou J. McBain C. Wright P. Knutson P. Armstrong R. (1996). Oligodendrocyte progenitor cell proliferation and lineage progression are regulated by glutamate receptor-mediated K^+^ channel block. J. Neurosci. 15, 2659–2670. 10.1523/JNEUROSCI.16-08-02659.1996 8786442 PMC6578780

[B33] Gibson J. Bartley A. Hays S. Huber K. (2008). Imbalance of neocortical excitation and inhibition and altered UP states reflect network hyperexcitability in the mouse model of fragile X syndrome. J. Neurophysiol. 100, 2615–2626. 10.1152/jn.90752.2008 18784272 PMC2585391

[B34] Gonçalves J. T. Anstey J. E. Golshani P. Portera-Cailliau C. (2013). Circuit level defects in the developing neocortex of Fragile X mice. Nat. Neurosci. 16, 903–909. 10.1038/nn.3415 23727819 PMC3695061

[B35] Guo W. Polich E. Su J. Gao Y. Christopher D. Am A.. (2015). Fragile X Proteins FMRP and FXR2P control synaptic GluA1 expression and neuronal maturation via distinct mechanisms. Cell Rep. 11, 1651–1666. 10.1016/j.celrep.2015.05.013 26051932 PMC4472556

[B36] Hagerman R. Hoem G. Hagerman P. (2010). Fragile X and autism: intertwined at the molecular level leading to targeted treatments. Mol. Autism 1, 1–14. 10.1186/2040-2392-1-12 20858229 PMC2954865

[B37] Hanse E. Seth H. Riebe I. (2013). AMPA-silent synapses in brain development and pathology. Nat. Rev. Neurosci. 14, 839–850. 10.1038/nrn3642 24201185

[B38] Harlow E. Till S. Russell T. Wijetunge L. Kind P. Contractor A. (2010). Critical period plasticity is disrupted in the barrel cortex of *FMR1* knockout mice. Neuron 65, 385–398. 10.1016/j.neuron.2010.01.024 20159451 PMC2825250

[B39] Harraza M. Eackera S. Wang X. Dawson T. Dawson V. (2012). MicroRNA-223 is neuroprotective by targeting glutamate receptors. Proc. Natl. Acad. Sci. U S A 109, 18962–18967. 10.1073/pnas.1121288109 23112146 PMC3503176

[B40] Hernandez R. Feinberg R. Vaurio R. Passanante N. Thompson R. Kaufmann E. (2009). Autism spectrum disorder in fragile X syndrome: a longitudinal evaluation. Am. J. Med. Genet. 149A, 1125–1137. 10.1002/ajmg.a.32848 19441123 PMC2734278

[B41] Hirai S. Hotta K. Kubo Y. Nishino A. Okabe S. Okamura Y.. (2017). AMPA glutamate receptors are required for sensory-organ formation and morphogenesis in the basal chordate. Proc. Natl. Acad. Sci. U S A 114, 3939–3944. 10.1073/pnas.1612943114 28348228 PMC5393200

[B42] Ho V. Dallalzadeh L. Karathanasis N. Keles M. Vangala S. Grogan T.. (2014). GluA2 mRNA distribution and regulation by miR-124 in hippocampal neurons. Mol. Cell. Neurosci. 61, 1–12. 10.1016/j.mcn.2014.04.006 24784359 PMC4134974

[B43] Huber K. Gallagher S. Warren S. Bear M. (2002). Altered synaptic plasticity in a mouse model of Fragile X mental retardation. Proc. Natl. Acad. Sci. U S A 99, 7746–7750. 10.1073/pnas.122205699 12032354 PMC124340

[B44] Hutchison E. R. Kawamoto E. M. Taub D. D. Lal A. Abdelmohsen K. Zhang Y.. (2013). Evidence for miR-181 involvement in neuroinflammatory responses of astrocytes. Glia 61, 1018–1028. 10.1002/glia.22483 23650073 PMC4624280

[B45] Jansson L. C. Louhivuori L. Wigren H. K. Nordström T. Louhivuori V. Castrén M. L.. (2013). Effect of glutamate receptor antagonists on migrating neural progenitor cells. Eur. J. Neurosci. 37, 1369–1382. 10.1111/ejn.12152 23383979

[B46] Jansson L. C. Wigren H. K. Nordström T. Akerman K. E. (2011). Functional α-amino-3-hydroxy-5-methylisoxazole-4-propionic acid receptors in differentiating embryonic neural progenitor cells. Neuroreport 22, 282–287. 10.1097/wnr.0b013e3283457b34 21399551

[B47] Jin P. Warren S. (2000). Understanding the molecular basis of fragile X syndrome. Hum. Mol. Genet. 9, 901–908. 10.1093/hmg/9.6.901 10767313

[B48] Kaufmann W. Kidd S. Andrews H. Budimirovic D. Esler A. Haas-Givler B.. (2017). Autism spectrum disorder in fragile X syndrome: cooccurring conditions and current treatment. Pediatrics 139, S194–S206. 10.1542/peds.2016-1159F 28814540 PMC5619699

[B49] Kim J. Park K. Kang R. Gonzales E. Kim D. Oh H.. (2019). Pharmacological modulation of AMPA receptor rescues social impairments in animal models of autism. Neuropsychopharmacology 44, 314–323. 10.1038/s41386-018-0098-5 29899405 PMC6300529

[B50] Kluger G. Böhm I. Laub M. Waldenmayer C. (1996). Epilepsy and fragile X gene mutations. Pediatr. Neurol. 15, 358–360. 10.1016/s0887-8994(96)00251-2 8972540

[B51] Kumar S. S. Bacci A. Kharazia V. Huguenard J. R. (2002). A developmental switch of AMPA receptor subunits in neocortical pyramidal neurons. J. Neurosci. 22, 3005–3015. 10.1523/JNEUROSCI.22-08-03005.2002 11943803 PMC6757523

[B52] Kwak S. Kawahara Y. (2005). Deficient RNA editing of GluR2 and neuronal death in amyotropic lateral sclerosis. J. Mol. Med. 83, 110–120. 10.1007/s00109-004-0599-z 15624111

[B53] La Fata G. Gärtner A. Domínguez-Iturza N. Dresselaers T. Dawitz J. Poorthuis R.. (2014). FMRP regulates multipolar to bipolar transition affecting neuronal migration and cortical circuitry. Nat. Neurosci. 17, 1693–1700. 10.1038/nn.3870 25402856

[B54] Lalanne T. Oyrer J. Mancino A. Gregor E. Chung A. Huynh L.. (2016). Synapse-specific expression of calcium-permeable AMPA receptors in neocortical layer 5. J. Physiol. 594, 837–861. 10.1113/jp271394 26537662 PMC4753277

[B55] Lauterborn J. Rex C. Kramár E. Chen L. Pandyarajan V. Lynch G.. (2007). Brain-derived neurotrophic factor rescues synaptic plasticity in a mouse model of fragile X syndrome. J. Neurosci. 27, 10685–10694. 10.1523/JNEUROSCI.2624-07.2007 17913902 PMC6672822

[B56] Lee R. Feinbaum R. Ambros V. (1993). The *C. elegans* heterochronic gene lin-4 encodes small RNAs with antisense complementarity to lin-14. Cell 75, 843–854. 10.1016/0092-8674(93)90529-y 8252621

[B57] Lewis B. P. Burge C. B. Bartel D. P. (2005). Conserved seed pairing, often flanked by adenosines, indicates that thousands of human genes are microRNA targets. Cell 120, 15–20. 10.1016/j.cell.2004.12.035 15652477

[B58] Li J. Pelletier M. Perez Velazquez J. Carlen P. (2002). Reduced cortical synaptic plasticity and GluR1 expression associated with fragile X mental retardation protein deficiency. Mol. Cell. Neurosci. 19, 138–151. 10.1006/mcne.2001.1085 11860268

[B59] Li Y. Zhao X. (2014). Concise review: fragile X proteins in stem cell maintenance and differentiation. Stem Cells 32, 1724–1733. 10.1002/stem.1698 24648324 PMC4255947

[B60] Lidow M. Rakic P. (1995). Neurotransmitter receptors in the proliferative zones of the developing primate occipital lobe. J. Comp. Neurol. 360, 393–402. 10.1002/cne.903600303 8543647

[B61] Lintas C. Sacco R. Persico A. (2012). Genome-wide expression studies in autism spectrum disorder, Rett syndrome and Down syndrome. Neurobiol. Dis. 45, 57–68. 10.1016/j.nbd.2010.11.010 21130877

[B62] Liu J. Koscielska K. A. Cao Z. Hulsizer S. Grace N. Mitchell G.. (2012). Signaling defects in iPSC-derived fragile X premutation neurons. Hum. Mol. Genet. 21, 3795–3805. 10.1093/hmg/dds207 22641815 PMC3412379

[B63] López T. López-Colomé A. Ortega A. (1994). AMPA/KA receptor expression in radial glia. Neuroreport 12, 504–506. 10.1097/00001756-199401120-00034 8003684

[B64] LoTurco J. Owens D. Heath M. Davis M. Kriegstein A. (1995). GABA and glutamate depolarize cortical progenitor cells and inhibit DNA synthesis. Neuron 15, 1287–1298. 10.1016/0896-6273(95)90008-x 8845153

[B65] Louhivuori V. Arvio M. Soronen P. Oksanen V. Paunio T. Castren M. L. (2009). The Val66Met polymorphism in the BDNF gene is associated with epilepsy in fragile X syndrome. Epilepsy Res. 85, 114–117. 10.1016/j.eplepsyres.2009.01.005 19394799

[B66] Malenka R. Nicoll R. (1997). Silent synapses speak up. Neuron 19, 473–476. 10.1016/s0896-6273(00)80362-1 9331339

[B67] Meredith R. Mansvelder H. (2010). STDP and mental retardation: dysregulation of dendritic excitability in fragile X syndrome. Front. Synaptic Neurosci. 2:10. 10.3389/fnsyn.2010.00010 21423496 PMC3059693

[B68] Meredith R. de Jong R. Mansvelder H. D. (2011). Functional rescue of excitatory synaptic transmission in the developing hippocampus in Fmr1-KO mouse. Neurobiol. Dis. 41, 104–110. 10.1016/j.nbd.2010.08.026 20817093

[B69] Meredith R. Holmgren C. Weidum M. Burnashev N. Huibert D. Mansvelder H. (2007). Increased threshold for spike-timing-dependent plasticity is caused by unreliable calcium signaling in mice lacking fragile X gene FMR1. Neuron 54, 627–638. 10.1016/j.neuron.2007.04.028 17521574

[B70] Mignogna M. Giannandrea M. Gurgone A. Fanelli F. Raimondi F. Mapelli L.. (2015). The intellectual disability protein RAB39B selectively regulates GluA2 trafficking to determine synaptic AMPAR composition. Nat. Comun. 6:6504. 10.1038/ncomms7504 25784538 PMC4383008

[B71] Nakamoto M. Nalavadi V. Epstein M. Narayanan U. Bassell G. Warren S. (2007). Fragile X mental retardation protein deficiency leads to excessive mGluR5-dependent internalization of AMPA receptors. Proc. Natl. Acad. Sci. U S A 104, 15537–15542. 10.1073/pnas.0707484104 17881561 PMC2000537

[B720] Olson H. E. Shen Y. Poduri A. Gorman M. Dies K. A. Robbins M.. (2012). Micro-duplications of 1q32.1 associated with neurodevelopmental delay. Eur. J. Med. Genetics. 55, 145–150. 10.1016/j.ejmg.2011.12.008 22266072 PMC3288188

[B72] O’Roak B. Vives L. Girirajan S. Karakoc E. Krumm N. Coe B.. (2012). Sporadic autism exomes reveal a highly interconnected protein network of *de novo* mutations. Nature 485, 246–250. 10.1038/nature10989 22495309 PMC3350576

[B73] Pachernegg S. Münster Y. Muth-Köhne E. Fuhrmann G. Hollmann M. (2015). GluA2 is rapidly edited at the Q/R site during neural differentiation *in vitro*. Front. Cell. Neurosci. 9:69. 10.3389/fncel.2015.00069 25798088 PMC4350408

[B74] Pardo C. Eberhart C. (2007). The neurobiology of autism. Brain Pathol. 17, 434–447. 10.1111/j.1750-3639.2007.00102.x 17919129 PMC8095519

[B75] Pellegrini-Giampietro D. E. Zukin R. S. Bennett M. V. Cho S. Pulsinelli W. A. (1992). Switch in glutamate receptor subunit gene expression in CA1 subfield of hippocampus following global ischemia in rats. Proc. Natl. Acad. Sci. U S A 89, 10499–10503. 10.1073/pnas.89.21.10499 1438239 PMC50366

[B76] Peng P. Zhong X. Tu W. Soundarapandian M. Molner P. Zhu D.. (2006). ADAR2-dependent RNA editing of AMPA receptor subunit GluR2 determines vulnerability of neurons in forebrain ischemia. Neuron 49, 719–733. 10.1016/j.neuron.2006.01.025 16504947

[B77] Persico A. Napolioni V. (2013). Autism genetics. Behav. Brain Res. 251, 95–112. 10.1016/j.bbr.2013.06.012 23769996

[B78] Pinto D. Pagnamenta A. Klei L. Anney R. Merico D. Regan R.. (2010). Functional impact of global rare copy number variation in autism spectrum disorders. Nature 466, 368–372. 10.1038/nature09146 20531469 PMC3021798

[B79] Purcell A. Jeon O. Zimmerman A. Blue M. E. Pevsner J. (2001). Postmortem brain abnormalities of the glutamate neurotrasmitter system in autism. Neurology 57, 1618–1628. 10.1212/wnl.57.9.1618 11706102

[B80] Ramanathan S. Woodroffe A. Flodman P. L. Mays L. Z. Hanouni M. Modahl C. B.. (2004). A case of autism with an interstitial deletion on 4q leading to hemizygosity for genes encoding for glutamine and glycine neurotransmitter sub-units (AMPA 2, GLRA 3, GLRB) and neuropeptide receptors NPY1R, NPY5R. BMC Med. Genet. 5:10. 10.1186/1471-2350-5-10 15090072 PMC411038

[B81] Richter J. D. Bassell G. J. Klann E. (2015). Dysregulation and restoration of translational homeostasis in fragile X syndrome. Nat. Rev. Neurosci. 16, 595–605. 10.1038/nrn4001 26350240 PMC4688896

[B82] Ruffolo D. Iyer A. Cifelli P. Rosetti C. Muhlebner A. Van Scheppingen J.. (2016). Functional aspects of early brain development are preserved in tuberous sclerosis complex (TSC) epileptogenic lesions. Neurobiol. Dis. 95, 93–101. 10.1016/j.nbd.2016.07.014 27425893

[B83] Rydzewska E. Hughes-Mccormack L. Gillberg C. Henderson A. Macintyre C. Rintoul J.. (2018). Prevalence of sensory impairments, physical and intellectual disabilities, and mental health in children and young people with self/proxy-reported autism: observational study of a whole country population. Autism [Epub ahead of print]. 10.1177/1362361318791279 30328695

[B84] Saba R. Störchel P. Aksoy-Aksel A. Kepura F. Lippi G. Plant T.. (2012). Dopamine-regulated microRNA MiR-181a controls GluA2 surface expression in hippocampal neurons. Mol. Cell. Biol. 32, 619–632. 10.1128/MCB.05896-11 22144581 PMC3266602

[B85] Saffary R. Xie Z. (2011). FMRP regulates the transition from radial glial cells to intermediate progenitor cells during neocortical development. J. Neurosci. 31, 1427–1439. 10.1523/jneurosci.4854-10.2011 21273427 PMC6623593

[B86] Santoro M. Bray S. M. Warren S. T. (2012). Molecular mechanisms of fragile X syndrome: a twenty-year perspective. Annu. Rev. Pathol. 7, 219–245. 10.1146/annurev-pathol-011811-132457 22017584

[B87] Schlett K. (2006). Glutamate as a modulator of embryonic and adult neurogenesis. Curr. Top. Med. Chem. 6, 949–960. 10.2174/156802606777323665 16787269

[B88] Ghahramani Seno M. M. Hu P. Gwadry F. G. Pinto D. Marshall C. R. Casallo G.. (2011). Gene and miRNA expression profiles in autism spectrum disorders. Brain Res. 1380, 85–97. 10.1016/j.brainres.2010.09.046 20868653

[B89] Sheridan S. D. Theriault K. M. Reis S. A. Zhou F. Madison J. M. Daheron L.. (2011). Epigenetic characterization of the *FMR1* gene and aberrant neurodevelopment in human induced pluripotent stem cell models of fragile X syndrome. PLoS One 6:e26203. 10.1371/journal.pone.0026203 22022567 PMC3192166

[B90] Siew W. Tan K. Babaei M. Cheah P. Ling K. (2013). MicroRNAs and intellectual disability (ID) in Down syndrome, X-linked ID and Fragile X syndrome. Front. Cell. Neurosci. 7:41. 10.3389/fncel.2013.00041 23596395 PMC3625835

[B91] Simonoff E. Pickles A. Charman T. Chandler S. Loucas T. Baird G. (2008). Psychiatric disorders in children with autism spectrum disorders: prevalence, comorbidity, and associated factors in a population-derived sample. J. Am. Acad. Child Adolesc. Psychiatry 47, 921–929. 10.1097/chi.0b013e318179964f 18645422

[B92] Soden M. E. Chen L. (2010). Fragile X protein FMRP is required for homeostatic plasticity and regulation of synaptic strength by retinoic acid. J. Neurosci. 30, 16910–16921. 10.1523/jneurosci.3660-10.2010 21159962 PMC3073636

[B93] Sommer B. Köhler M. Sprengel R. Seeburg P. (1991). RNA editing in brain controls a determinant of ion flow in glutamate-gated channels. Cell 67, 11–19. 10.1016/0092-8674(91)90568-j 1717158

[B94] Spaethling J. M. Klein D. M. Singh P. Meaney D. F. (2008). Calcium-permeable AMPA receptors appear in cortical neurons after traumatic mechanical injury and contribute to neuronal fate. J. Neurotrauma 25, 1207–1216. 10.1089/neu.2008.0532 18986222 PMC2799682

[B95] Stepniak B. Kästner A. Poggi G. Mitjans M. Begemann M. Hartmann A.. (2015). Accumulated common variants in the broader fragile X gene family modulate autistic phenotypes. EMBO Mol. Med. 7, 1565–1579. 10.15252/emmm.201505696 26612855 PMC4693501

[B96] Suvrathan A. Hoeffer C. Wong H. Klann E. Chattarji S. (2010). Characterization and reversal of synaptic defects in the amygdala in a mouse model of fragile X syndrome. Proc. Natl. Acad. Sci. U S A 107, 11591–11596. 10.1073/pnas.1002262107 20534533 PMC2895119

[B97] Szczurowska E. Ergang P. Kubova H. Druga R. Salaj M. Mares P. (2016). Influence of early life status epilepticus on the developmental expression profile of the GluA2 subunit of AMPA receptors. Exp. Neurol. 283, 97–109. 10.1016/j.expneurol.2016.05.039 27288240

[B98] Talos D. Fishman R. Park H. Folkerth R. Follett P. Volpe J.. (2006). Developmental regulation of α-amino-3-hydroxy-5-methyl-4-isoxazole-propionic acid receptor subunit expression in forebrain and relationship to regional susceptibility to hypoxic/ischemic injury. I. Rodent cerebral white matter and cortex. J. Comp. Neurol. 497, 42–60. 10.1002/cne.20972 16680782 PMC4313670

[B99] Telias M. Kuznitsov-Yanovsky L. Segal M. Ben-Yosef D. (2015). Functional deficiencies in fragile X neurons derived from human embryonic stem cells. J. Neurosci. 35, 15295–15306. 10.1523/jneurosci.0317-15.2015 26586818 PMC6605488

[B100] Tervonen T. A. Louhivuori V. Sun X. Hokkanen M. E. Kratochwil C. F. Zebryk P.. (2009). Aberrant differentiation of glutamatergic cells in neocortex of mouse model for fragile X syndrome. Neurobiol. Dis. 33, 250–259. 10.1016/j.nbd.2008.10.010 19056494

[B101] Tran S. Jun H. Bahn J. Azghadi A. Ramaswami G. Van Nostrand E.. (2019). Widespread RNA editing dysregulation in brains from autistic individuals. Nat. Neurosci. 22, 25–36. 10.1038/s41593-018-0287-x 30559470 PMC6375307

[B102] Uzunova G. Hollander E. Shepherd J. (2014). The role of ionotropic glutamate receptors in childhood neurodevelopmental disorders: autism spectrum disorders and fragile x syndrome. Curr. Neuropharmacol. 12, 71–98. 10.2174/1570159x113116660046 24533017 PMC3915351

[B103] Wang H. Kim S. Zhuo M. (2010). Roles of fragile X mental retardation protein in dopaminergic stimulation-induced synapse-associated protein synthesis and subsequent α-amino-3-hydroxyl-5-methyl-4-isoxazole-4-propionate (AMPA) receptor internalization. J. Biol. Chem. 285, 21888–21901. 10.1074/jbc.m110.116293 20457613 PMC2898437

[B104] Verkerk A. Pieretti M. Sutcliffe J. Fu Y.-H. Kuhl D. P. Pizzuti A.. (1991). Identification of a gene (FMR-1) containing a CGG repeat coincident with a breakpoint cluster region exhibing length variation in fragile X syndrome. Cell 65, 905–914. 10.1016/0092-8674(91)90397-h 1710175

[B105] Whitehead G. Regan P. Whitcomb D. J. Cho K. (2017). Ca^2+^-permeable AMPA receptor: a new perspective on amyloid-β mediated pathophysiology of Alzheimer’s disease. Neuropharmacology 112, 221–227. 10.1016/j.neuropharm.2016.08.022 27561971

[B106] Whitney N. P. Peng H. Erdmann N. B. Tian C. Monaghan D. T. Zheng J. C. (2008). Calcium-permeable AMPA receptors containing Q/R-unedited GluR2 direct human neural progenitor cell differentiation to neurons. FASEB J. 22, 2888–2900. 10.1096/fj.07-104661 18403631 PMC2493446

[B107] Wong S. K. Sato S. Lazinski D. W. (2001). Substrate recognition by ADAR1 and ADAR2. RNA 7, 846–858. 10.1017/s135583820101007x 11421361 PMC1370134

[B108] Wright A. Vissel B. (2012). The essential role of AMPA receptor GluR2 subunit RNA editing in the normal and diseased brain. Front. Mol. Neurosci. 5:34. 10.3389/fnmol.2012.00034 22514516 PMC3324117

[B109] Wu G. Malinow R. Cline H. T. (1996). Maturation of a central glutamatergic synapse. Science 274, 972–976. 10.1126/science.274.5289.972 8875937

[B110] Xu Z. Yang Q. Ma L. Liu S. Chen G. Wu Y.. (2012). Deficits in LTP induction by 5-HT2A receptor antagonist in a mouse model for fragile X syndrome. PLoS One 7:e48741. 10.1371/journal.pone.0048741 23119095 PMC3485341

[B111] Yang Z. Wan X. Gu Z. Zhang H. Yang X. He L.. (2014). Evolution of the miR-181 microRNA family. Comput. Biol. Med. 52, 82–87. 10.1016/j.compbiomed.2014.06.004 25016292

[B112] Zhang Y. Bonnan A. Bony G. Ferezou I. Pietropaolo S. Ginger M.. (2014). Dendritic channelopathies contribute to neocortical and sensory hyperexcitability in Fmr1(-/y) mice. Nat. Neurosci. 17, 1701–1709. 10.1038/nn.3864 25383903

[B113] Zhao M. G. Toyoda H. Ko S. W. Ding H. K. Wu L. J. Zhuo M. (2005). Deficits in Trace Fear Memory and long-term potentiation in a mouse model for fragile X syndrome. J. Neurosci. 25, 7385–7392. 10.1523/jneurosci.1520-05.2005 16093389 PMC6725289

[B114] Zonouzi M. Renzi M. Farrant M. Cull-Candy S. G. (2011). Bidirectional plasticity of calcium-permeable AMPA receptors in oligodendrocyte lineage cells. Nat. Neurosci. 14, 1430–1438. 10.1038/nn.2942 21983683 PMC3204222

